# Acute Kidney Injury, Renal Replacement Therapy, and Extracorporeal Membrane Oxygenation Treatment During the COVID-19 Pandemic: Single-Center Experience

**DOI:** 10.3390/medicina61020237

**Published:** 2025-01-28

**Authors:** Fabrizio Ceresa, Paolo Monardo, Antonio Lacquaniti, Liborio Francesco Mammana, Aurora Leonardi, Francesco Patanè

**Affiliations:** 1 Cardio-Vascular and Thoracic Department, Papardo Hospital, 98158 Messina, Italy; 2 Nephrology and Dialysis Unit, Papardo Hospital, 98158 Messina, Italy

**Keywords:** acute kidney injury (AKI), extracorporeal membrane oxygenation support (ECMO), acute respiratory distress syndrome (ARDS)

## Abstract

*Background and Objectives*: Severe acute respiratory syndrome coronavirus 2 (SARS-CoV2) was described in December 2019 for the first time, and it was responsible for a global pandemic. An alarming number of patients with coronavirus disease 2019 (COVID-19) also developed acute kidney injury (AKI), especially those who required extracorporeal membrane oxygenation (ECMO) therapy for acute respiratory distress syndrome (ARDS). The aim of our retrospective observational study was to assess the prognostic significance of AKI in these patients. This study observed, in COVID-19 patients admitted to an intensive care unit (ICU), AKI stages and the need for renal replacement therapy (RRT), assessing the risk factors and outcomes. Moreover, we evaluated the mortality rate of patients treated by ECMO. *Materials and Methods*: Between November 2020 and December 2022, among 396 patients admitted to our intensive care unit (ICU) diagnosed with SARS-CoV-2 infection, we selected patients with severe ARDS requiring veno-venous (vv) ECMO support and AKI. *Results:* The 30-day mortality after ECMO positioning was 85.7%. A Cox regression revealed a significant advantage for RRT with a high cut-off (HCO) hemofilter both for ICU mortality (HR 0.17 [95% CI: 0.031–0.935], *p* = 0.035) and 15 day-mortality after the start of vv-ECMO (HR 0.13 [95%CI: 0.024–0.741], *p*= 0.021), whereas the early onset of vasoplegic shock after ECMO implantation indicated a higher risk of death (HR 11.55 [95% CI: 1.117–119.567], *p* = 0.04) during the ICU stay. *Conclusions:* COVID-19 induces a high risk of AKI and RRT. In our cohort, hypertension, pre-existing renal disease, and mechanical ventilation represented independent risk factors for AKI. Patients requiring ECMO support had a high mortality rate. The early implementation of RRT reduced the risk of death during the ICU stay.

## 1. Introduction

Severe acute respiratory syndrome coronavirus 2 (SARS-CoV 2) was described in December 2019 for the first time, was responsible for a global pandemic, and was officially named “coronavirus disease 2019” (COVID-19), becoming a worldwide emergency and leading to the collapse of the public health system [1].

As of March 2022, there have been more than 470 million COVID-19 cases and more than 6 million deaths from COVID-19 worldwide.

An alarming number of patients with COVID-19 also developed acute kidney injury (AKI), ranging from 6.5% to 57.4% [2,3,4], especially in the most critically ill (23–81%), and it was associated with a higher mortality rate [5,6].

Although the pathophysiology of AKI related to COVID-19 is unclear, several mechanisms have been suggested based on a direct and diffuse tubular cell injury mediated by the link between the virus and the angiotensin-converting enzyme 2 receptor, which is highly expressed in the renal proximal tubular cells and podocytes [7]. Moreover, COVID-19 causes a hyper-inflammatory systemic response due to excessive cytokine release, which includes cytokines such as interleukin-6 (IL-6), leading to intrarenal inflammation, increased vascular permeability, and alterations in kidney microcirculation [8].

The lung–kidney crosstalk, rhabdomyolysis, renal hypoperfusion, and nephrotoxic effects of mechanical ventilation represent other potential mechanisms linking infection with the virus to AKI [9]. Patients requiring both renal replacement therapy (RRT) and invasive ventilation had the highest in-hospital mortality rate of 73% in COVID-19 patients [10].

Behind renal damage, COVID-19 pneumonia caused the development of acute respiratory distress syndrome (ARDS), characterized by severe hypoxemia, often refractory to ventilator management and requiring, in selected patients, veno-venous (VV) extracorporeal membrane oxygenation (ECMO) as a rescue therapy [11,12,13,14].

AKI in patients with ECMO is reported in 26–85% of patients, with a high variation in reported incidences attributable to different clinical settings and patient characteristics, and RRT is strongly associated with a high mortality risk [15,16].

This study aimed to observe, in a cohort of COVID-19 patients admitted to the ICU, the phenomenon of AKI. We evaluated its stages and the need for RRT, assessing the risk factors and outcomes. Moreover, we also analyzed a subgroup of patients treated with ECMO therapies, evaluating the coexistence, or not, of AKI. The mortality rate of this subgroup and the entire cohort was also described.

### 1.1. Study Population

Between November 2020 and December 2022, we retrospectively enrolled 396 adult patients admitted to intensive care unit (ICU) beds dedicated to COVID-19 patients at the Papardo Hospital in Messina (Italy) due to acute respiratory failure secondary to SARS-CoV-2 infection. In particular, patients presenting an oxygen peripheral saturation (SpO_2_) lower than 85% and dyspnea were eligible for ICU admission.

All patients underwent nasopharyngeal swab collection for real-time polymerase chain reaction (RT-PCR) testing to detect SARS-CoV-2, and all infected patients were included in this study. Conversely, patients with end-stage renal disease or kidney transplants and acute neurological injury with poor prognosis were not included in this study. Moreover, we excluded from the enrollment patients with a long invasive mechanical duration of >10 days, the inability to receive systemic anticoagulation, ongoing cardiopulmonary resuscitation, age > 65 years, and oncologic and immunocompromised subjects.

Patients with severe multiorgan failure at ICU admission, defined as a sequential organ failure assessment (SOFA) score of >8, were also excluded from this study due to the high risk of mortality [17].

ARDS was defined according to the Berlin definition, using the PaO_2_/FiO_2_ ratio (Horowitz index) as a marker for severity [18].

AKI, according to the Kidney Disease Improving Global Outcomes (KDIGO) criteria, was diagnosed when the serum creatinine (sCr) level increased by 0.3 mg/dL within 48 h or the known or presumed sCr increased until 1.5 times the baseline value within the preceding 7 days, and urine output less was than 0.5 mL/kg/h for 6 h [19].

Moreover, RRT was started by the attending nephrologist according to local standardized protocols.

In particular, the modality of the prescribed RRT was continuous veno-venous hemodialysis (CVVHD), and all procedures were performed using PrisMax (Baxter Healthcare Corporation, Chicago, IL, USA) and Multifiltrate (Fresenius Medical Care, Deutschland) monitors, associated with oxiris and emic-2 hemofilters, respectively.

Citrate was used as the loco-regional circuit anticoagulation method in all RRT patients.

RRT in patients with vv-ECMO was performed via a separate central venous access. A sepsis diagnosis was based on the Sepsis-3 criteria: documented infection (infection signs and antibiotic treatment for more than 48 h) plus an increase in the acute SOFA score of 2 points or more [20].

At ICU admission, sera samples were collected for biomarker profiling evaluation and measured at the Papardo Hospital laboratory through standard protocols in a technician-blinded manner. In particular, the infective status was evaluated by white blood cell count (WBC) and C reactive protein (CRP), such as hepatic and renal function. Moreover, the evaluation of interleukin-6 reflected the inflammation status of the patients.

We selected the patients to treat with vv-ECMO according to the ELSO Guidelines [21] as follows: PaO_2_/FiO_2_ < 80 mmHg for >6 h; PaO_2_/FiO_2_ < 50 mmHg for >3 h; pH < 7.25 with PaCO_2_ > 60 mmHg for >6 h. A Cardiohelp (Getinge, Göteborg, Sweden) was used for all patients. Both dual- and single-site cannulation were indifferently used. When single-site cannulation was performed, an Avalon cannula was used. All patients received intravenous unfractionated heparin (UFH) bolus of 5000 units before initiation of vv-ECMO support. Afterward, UFH was administrated at the initial dose of 10 UI/Kg/h titrating the infusion rate to maintain an activated partial thromboplastin time (aPTT) between 45 and 60 s.

Before vv-ECMO started, all patients with PaO_2_/FiO_2_ < 150 mmHg underwent conventional treatment options established for ARDS, including lung-protective mechanical ventilation, neuromuscular blockade, and prone positioning [22].

### 1.2. Statistical Analysis

Statistical analyses were performed with SPSS 24 (SPSS Inc, Chicago, IL, USA) software and the GraphPad Prism (version 9.4.1; GraphPad Software, Inc., San Diego, CA, USA) package.

Data are presented as mean ± SD for normally distributed values and median (range) for non-normally distributed values according to the Kolmogorov–Smirnov test.

Differences between groups were established by an unpaired t-test or ANOVA followed by Bonferroni’s test for normally distributed values and by Kruskal–Wallis analysis followed by Dunn’s test for nonparametric values. Independent risk factors of AKI stages 2 and 3 were assessed using multivariable logistic regression analysis. Variables associated with AKI stages 2 and 3 in univariate analysis were included in the model. To predict 30-day mortality, multivariate logistic regression was performed for the binary variables and univariate linear regression was performed for the continuous variables.

Kaplan–Meier curves were generated to assess the survival in patients treated by ECMO and RRT. Differences were evaluated using the log-rank test. Adjusted risk estimates for death during an ICU stay were calculated using univariate followed by multivariate Cox proportional hazard regression analysis. Statistical significance was considered if *p*-value < 0.05.

This study was conducted in accordance with the Declaration of Helsinki and approved by the Ethics Committee of the University of Messina (Protocol code 41-24; Approval Date: 8 October 2024).

## 2. Results

### 2.1. Baseline Patient Characteristics

A total of 396 COVID-19 patients were included in our cohort. The characteristics of the entire cohort studied are summarized in Table 1.

The mean age of the patients was 57.6  ±  7.8 years, and 67% of them were females. The mean body mass index was 24.7  ±  4.6  kg/m^2^. Overall, 31.% of the patients were smokers, whereas 80% of the patients suffered from arterial hypertension (*n*: 315), 18% from diabetes mellitus type II (*n*: 71), 6% from chronic lung disease (*n*: 28), 24% chronic kidney disease (CKD, *n*: 98), and 20% chronic ischemic heart disease (*n*: 80).

All patients underwent thoracic computerized tomography, revealing radiological signs of COVID-19-related infection in 348 patients (88%). In particular, the interstitial involvement was <30% in 116 patients, between 30 and 50% in 87 patients, between 50 and 70% in 66 patients, and >70% in 79 patients.

The mean SOFA score at ICU admission was 5.7 ± 0.9, whereas the median duration of ICU stay was 18 (IQR 9–32) days. Overall, 36% of the patients received non-invasive ventilation, whereas the remaining 64% required invasive mechanical ventilation. In total, 140 (35%) patients underwent RRT during their ICU stay, and ECMO was used in 10% of cases.

The overall mortality was 32% (*n*: 126).

### 2.2. AKI Association with RRT Among COVID-19 Patients

AKI occurred in 134 (34%) patients, and RRT was applied during their ICU stay secondary to sepsis/septic shock in 86% of the cases. The mean values of serum creatinine at ICU admission and peak were, respectively, 1.06 ± 0.87 mg/dL and 2.41 ± 0.87 mg/dL.

The mean time of RRT initiation was 4.6 ± 2.4 days from ICU admission and was strongly associated with mortality, highlighting that 69 out of 140 (49%) RRT patients died. Moreover, AKI patients were older and affected by several concomitant diseases, such as pre-existing CKD, coronary artery disease, blood hypertension, and diabetes.

Univariate analysis showed that pre-existing CKD, diabetes mellitus, hypertension, coronary artery disease, and invasive mechanical ventilation were associated with severe AKI (*p* < 0.1). Based on the multivariate analysis, the independent risk factors for AKI were hypertension (odds ratio (OR): 1.16, CI: 1.01–3.22), pre-existing CKD (OR: 2.32, CI: 1.87–4.68), and mechanical ventilation (OR: 5.16, CI: 3.71–7.98); (*p* < 0.1).

### 2.3. ECMO and RRT Among COVID-19 Patients

The total number of patients with severe ARDS requiring vv-ECMO therapy was 41 (10%).

In this cohort, the frequency of COPD was less than in the overall population of patients with COVID-19 admitted to the ICU, whereas about 43% of them suffered from obesity.

Age, sex, and other baseline characteristics were the same as the overall enrolled population. The mean duration time in the ICU was 17.53 ± 14.82 days, whereas the mean duration of vv-ECMO therapy was 16.71 ± 11.63 days.

A SOFA score of 7.85 ± 3 was revealed at the start of ECMO support.

We did not record right ventricular dysfunction, defined as TAPSE < 14 mm, or tricuspid regurgitation (TR) degree ≥ 2+, within 30 days after receiving vv-ECMO (TAPSE: 20.5 ± 0.5 mm: TR ≥ 2+ = 0%).

A total of 29 patients (71%) required RRT, which was started at the same time as ECMO therapy. In particular, 14% were in KDIGO Stage 1, 29% in Stage 2, and 57% in Stage 3. The patients who did not receive RRT treatment maintained a urinary output >1 mg/Kg/h during all ICU stays, with a valid fluid balance and without severe pH alteration or severe electrolyte abnormalities.

**Figure 1:**
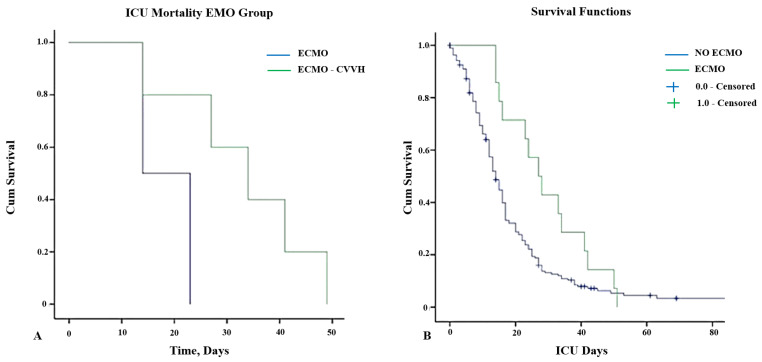
(**A**) Kaplan–Meier curves for ICU survival in patients receiving vv-ECMO therapy stratified according to the use of CVVH (log-rank = 0.01). (**B**) Kaplan–Meier curves for overall patient survival stratified according to the use of vv-ECMO support and censored for discharge from the ICU (log-rank = 0.031). Abbreviations: ICU: intensive care unit; ECMO: extracorporeal membrane oxygenation; CVVH: continuous veno-venous hemodialysis.

The 15-day and 30-day ICU mortalities after ECMO started were 57.1% and 85.7%, respectively, whereas the 30-day ICU mortality was 100%. Figure 1 illustrates the survival curves for patients treated by ECMO alone and associated with RRT.

All causes of death were multiorgan failure and septic shock caused by a secondary bacterial infection. In particular, in all patients, death occurred with negative tests for COVID-19 infection, whereas bacteria were isolated in all hemocultures and/or bronchoalveolar lavage cultures. *Pseudomonas aeruginosa*, *Acinetobacter baumannii*, *Staphylococcus aureus*, and *Klebsiella pneumoniae* were the most frequent bacteria isolated, whereas the most common antimicrobial agents were beta-lactam/beta-lactamase inhibitors (44%), macrolides (21%), cephalosporins (13%), and carbapenems (9%).

Based on the logistic regression, we did not find an independent risk factor for 30-day mortality in the ICU.

The Cox regression with an interaction term for ICU mortality, RRT, and vv-ECMO therapy revealed a significant advantage for RRT both for ICU mortality (HR 0.17 [95% CI: 0.031–0.935], *p* = 0.035) and 15-day mortality after the start of vv-ECMO (HR 0.13 [95%CI: 0.024–0.741], *p*= 0.021). The Cox regression for a time until death in the ICU for these patients revealed that the onset of vasoplegic shock, defined as the need for norepinephrine >0.2 μg/kg/min to maintain a mean arterial pressure (MAP) >65 mmHg for 3 days post-ECMO implantation indicates a higher risk of death (HR 11.55 [95% CI: 1.117–119.567], *p* = 0.04).

Inflammatory markers were modified by vv-ECMO and RRT (group 1) if compared to levels observed in patients treated by vv-ECMO alone (group 2). In particular, C reactive protein and interleukin-6 levels were significantly lower in group 1 than in group 2 (*p* = 0.0001) at 1, 3, and 7 days, as well as the changes in the white blood cell (WBC) count (WBC at 1 d vs. WBC at 3 d: *p* = 0.019; WBC at 1 d vs. WBC at 7 d: *p* = 0.011), as shown in Table 2.

The increase in total bilirubin (TB) levels (TB 1 day vs. TB 3 day: *p* = 0.002; TB 1 day vs. TB 7 day: *p* = 0.002: TB 3 day vs. TB 7 day: *p* = 0.009) and liver enzyme values (AST 1 day vs. AST 7 day: *p* = 0.014; AST 3 day vs. AST 7 day: *p* = 0.001) were not related to the type of venous cannulation for ECMO (single site—Avalon—vs. double site—standard cannula; TB: *p* = 0.251; liver enzymes: *p* = 0.238). Table 3.

## 3. Discussion

Our data revealed that severe AKI requiring RRT complicated the clinical course of critically ill COVID-19 patients.

Generally, the overall incidence of COVID-19 AKI in the patients admitted to the ICU was 34%, a slightly lower value than the data published, which ranged from 50% to 81% [23]. Pulmonary manifestations of COVID-19 are the most common, but kidney injury is not a rare complication, and it is evident at hospital admission. Mortality among hospitalized COVID-19 patients associated with AKI is higher than those without kidney involvement.

CKD is a well-known risk factor for AKI in hospitalized patients. Several epidemiological studies identified CKD as a relevant and independent risk factor for worse outcomes in COVID-19. However, in our cohort, only 24% of patients suffered from CKD.

Although the reported incidence and severity of AKI in COVID-19 patients depend on the clinical setting and the definitions used, some studies reported an incidence that achieves up to 78% in those requiring intubation. We corroborated these data, revealing that mechanical ventilation represents an independent risk factor for AKI development.

Endothelial dysfunction and the coagulation cascade also play an important role in the pathophysiology of COVID-19 AKI. Several biomarkers of coagulation and fibrinolysis were altered in these patients, as well as the increased levels of biomarkers of endothelial damage and platelet activation associated with poor prognosis [24,25]. Furthermore, patients with severe COVID-19 have chronic endothelial dysfunction due to several underlying co-morbidities, such as hypertension or diabetes, which were independent risk factors for AKI.

The release of inflammatory mediators by immune and resident kidney cells represents a mechanism of tissue damage in patients with COVID-19. Inflammatory mediators, by binding their specific receptors expressed by renal endothelial and tubule epithelial cells, cause a direct injury [26,27]. The complement cascade is usually involved in the innate immune response to viral infections, even if its persistent and uncontrolled activation can promote inflammatory processes leading to tissue injury.

The inflammatory response of COVID-19 bears similarities to the other conditions that are associated with cytokine storm syndrome (CSS). CSS is a life-threatening condition characterized by organ failure and the rapid proliferation and hyperactivity of all immune system components, including T cells, macrophages, natural killer cells, and the increased production and release of inflammatory cytokines.

Indeed, in the patients with COVID-19, IL-6 levels were elevated, suggesting hyperactivation of the humoral immune response. It has been demonstrated that IL-6 is a critical mediator of multiorgan dysfunction, including AKI, and its high levels are associated with adverse clinical outcomes and mortality.

Although the observed levels of IL-6 in severe COVID-19 are lower than in septic shock or the hyper-inflammatory response, its involvement in the regional inflammation in the pathogenicity of COVID-19 has been supported by the literature data.

The interaction between the cardiovascular system and the kidney is also likely to contribute to COVID-19 AKI.

As in another form of ARDS, the increase in intrathoracic pressure, right atrial pressure, and right ventricular afterload depends on the use of high positive end-expiratory pressure and/or tidal volumes. Right-side dysfunction leads to increased venous pressure, causing a reduction in GFR and oxygen delivery to the kidney as a consequence of an increased interstitial and tubular hydrostatic pressure.

Moreover, following the development of AKI, increases in the levels of inflammatory cytokines, such as IL-6, also depend on its reduced renal clearance and may contribute to respiratory failure via kidney–lung crosstalk.

In critically ill COVID-19 patients requiring organ circulatory support, the role of ECMO in the development of AKI is still debated.

The initiation of ECMO is associated with an immediate and complex inflammatory reaction due to the contact between the blood and the surface of the circuit, which promotes the activation of coagulative and inflammatory cascades.

Several associated plasma proteins, such as factor XII, were activated within 10 minutes of ECMO initiation, promoting coagulation and increasing the bradykinin levels.

Afterward, activation of the intrinsic and extrinsic coagulation pathways determines an overproduction of thrombin, inducing thrombotic inflammation effects and endothelial dysfunction, promoting the expression of P-selectin proteins in endothelial cells, and activating neutrophils and platelets.

Moreover, the pro-thrombotic effects observed in COVID-19 patients requiring vv-ECMO support could cause some problems in the management of anticoagulation. The anti-Xa assay, bivalirudin, or nafamostat mesylate are proposed as an anticoagulation strategy to better balance the risk of bleeding and thrombosis [28,29,30].

The complement and cytokine systems are also involved in endothelium injury during ECMO. IL-6 belongs to the pro-inflammatory cytokines, playing an important role in initiating the acute phase response, and its levels are very high during ECMO. Several studies demonstrated that IL-6 levels are inversely associated with survival in these patients [31].

Thus, COVID-19-related AKI seems to depend on both virus-specific and non-specific factors, contributing to amplifying endothelial dysfunction and cytokine storm.

In clinical practice, some studies showed that in patients with severe COVID-19, CRRT timing is very important as it can improve the prognosis and reduce the mortality rate by blocking the activation of the inflammatory cascade within 72 hours [32].

Regarding the requirement for ECMO support, the efficacy of RRT is still controversial. ECMO is usually the last chance for severe ARDS related to COVID-19, and its benefit has been debated since the onset of the pandemic.

COVID-19 infection, characterized by a severe inflammatory response if ECMO is administered, induces the release of cytokines and leads to a worsening of inflammation. However, ECMO remains a life-threatening therapy for these patients.

Hence, the combination of vv-ECMO and RRT could be an interesting option, which allowed for a reduction in inflammatory factors and the ground-glass opacity at the computed tomography scan of the lung.

The usefulness of RRT with a high cut-off (HCO) hemofilter in COVID-19-related AKI is another debated topic, removing the most cytokines and reducing the inflammatory response. HCO hemofiltration improved cytokine clearance in patients with septic shock, which was associated with an improvement in hemodynamic condition, oxygenation indices, and organ dysfunction [33].

Different types of HCO filters are available, such as Septex, Oxiris, and EMiC2. We used an EMiC2 hemofilter in all patients with ECMO support undergoing RRT. Several studies published opposing results about RRT with EMiC2 in septic shock. A significant reduction in IL-6 values, especially after 24–36 h of treatment, was recorded, whereas other results underlined the inefficacy over 72 hours. Other data refer to cytokine modulation and impacts on hemodynamic stability, but the results in severely ill COVID-19 patients are debated.

Cytosorb (CytoSorbents, Monmouth Junction, NJ, USA) represents another adsorber hemofilter containing hemocompatible porous polymer chains capable of reducing molecules of medium weight (5–55 kDa), such as cytokines, toxins, and therapeutic drugs, from the blood. Although data on clinical effectiveness are inconsistent, Cytosorb is widely used in septic patients. The absorber can easily be integrated into extracorporeal blood circulation devices such as hemodialysis or ECMO.

The current results are discordant in terms of its advantage in critically ill COVID-19 patients [31,34,35].

Two randomized controlled trials investigated the effect of Cytosorb in these patients, revealing higher or no effects on mortality in patients treated with ECMO and Cytosorb [23,36].

Conversely, in septic shock, extracorporeal cytokine adsorption that was started less than 24 hours from ICU admission led to a significant reduction in vasoconstrictor requirements and inflammatory mediator levels. Although our study included only patients with severe COVID-19 requiring VV-ECMO therapy, our results have not been confirmed by published data [23].

Although there are several studies that assessed the results of hemoperfusion and blood purification strategies in patients with COVID-19, the most recent data seem to not strongly support their clinical efficacy, even in addition to ECMO support.

In our experience, the early implementation of RRT with an HCO hemofilter in critically ill COVID-19 patients requiring ECMO support can reduce the 30-day ICU mortality, without effects on overall mortality.

In other studies, authors found that the development of AKI in these patients significantly increases ICU mortality.

Also, the timing of implanting vv-ECMO support may influence the prognosis, as pointed out by Pans, who observed that patients with an arterial pH lower than 7.25 before initiation of ECMO had a worse prognosis [21]. This finding suggests that ECMO should be used early before respiratory failure due to decreased lung compliance as compared to patients in whom ECMO was still initiated for oxygenation failure.

Several studies observed that lung function recovery in COVID-19 patients is slow and the weaning of ECMO could be delayed, even if this prolonged support did not seem to worsen the outcome. On the contrary, a large meta-analysis found that ECMO duration was associated with increased mortality, probably due to the onset of complications in a time-dependent manner.

The most common complication was major bleeding, requiring an increased number of blood transfusions and worsening lung inflammation.

Indeed, some studies demonstrated difficulty in balancing the risks of bleeding and clotting in these patients due to the alterations of the coagulative cascade related to COVID-19.

Another important risk factor of ICU mortality is right ventricular involvement [37].

In fact, in the case of right ventricular dysfunction, Mustafa showed a good result using a ProTek duo cannula in the vv ECMO setting, upgrading the circulatory support and achieving a right ventricular assist device [38].

Generally, the first results of ECMO therapy, especially during the first wave of the pandemic, were very discouraging, reporting a very high mortality rate of up to 83%. After understanding the mechanism of COVID-19 infection better, further studies revealed an improvement in outcomes, finding a mortality rate of patients with vv-ECMO that ranged from 31% to 58.9% [39].

In our experience, the 30-day mortality in patients with severe AKI who received ECMO support remains very high, according to other studies [34], especially in case of the early onset of vasoplegic shock after the start of ECMO.

The early implementation of RRT with an HCO hemofilter (EMiC2) led to a lower risk of death during the ICU stay in these patients, although the overall mortality did not improve.

The present study has several limitations that should be underlined. First, this was a retrospective observational study conducted at a single center and is thus subject to the inherent errors associated with this methodology. Moreover, we excluded patients older than 65 years and with prolonged mechanical ventilation, reducing confounding factors. Furthermore, we did not separate the patients in relation to the pandemic waves they belong to, and this could be a bias. We speculated that the values of inflammatory and infective markers could be related to the effect of RRT associated with ECMO, but these results need to be confirmed in wider cohorts to attribute general validity to our reports.

## 4. Conclusions

In conclusion, COVID-19 induces a high risk of AKI and RRT. In our cohort, hypertension, pre-existing CKD, and mechanical ventilation represented independent risk factors for AKI. These patients required ECMO support with a high mortality rate. The early implementation of RRT with an HCO hemofilter reduced the risk of 30-day mortality during the ICU stay.

## Figures and Tables

**Table 1:** Clinical parameters of enrolled COVID-19 patients.

	All Patients *n*: 396
Age, years	57.6 ± 7.8
BMI, kg/m^2^	24.7 ± 4.6
Hypertension, n (%)	315 (80)
Smoke, n (%)	123 (31)
Diabetes n (%)	71 (18)
COPD n (%)	28 (6)
CKD n (%)	98 (24)
Ischemic heart disease	80 (20)
SOFA	5.7 ± 0.9
RRT n (%)	140 (35)
ECMO n (%)	41 (10)
Mortality, n (%)	126 (32)

Abbreviations: BMI: body mass index; COPD: chronic obstructive pulmonary disease; CKD: chronic kidney disease; SOFA: sequential organ failure assessment; ECMO: extracorporeal membrane oxygenation.

**Table 2:** Inflammatory markers in ECMO patients treated or not by RRT.

		RRT			No RRT	
	Day 1	Day 3	Day 7	Day 1	Day 3	Day 7
IL-6 (ng/L)	78.0 ± 97.1	438.2 ± 562.3	358.5 ± 606.2 a	34.3 ± 0.2	202.5 ± 3.5	203.5 ± 4.9 #
CRP (mg/dL)	21.6 ± 12.5	20.2 ± 14.9	18.3 ± 9 b	41.95 ± 13.4	45.85 ± 2.1	58.45 ± 13.2 b
WBC (×10^3^ U/L)	20.9 ± 4.2 a	18.9 ± 12.4 a	14.3 ± 11 c	49.8 ± 40.6 a	29.9 ± 27.8 a	32.5 ± 24.7 c

Abbreviations: ECMO: extracorporeal membrane oxygenation; RRT: renal replacement therapy; IL-6: interleukin-6; CRP: C-reactive protein; WBC: white blood cell; ALT: alanine transaminase; AST: aspartate transaminase; TB: total bilirubin level. a: *p* = 0.0001 day 3 vs. day 7 in the RRT group; b: *p* = 0.0001 day 1 vs. day 7 in the RRT group and the no RRT group; c: *p* = 0.01 day 1 vs. day 7 in the RRT group and the no RRT group; **#**: *p*> 0.05 day 3 vs. day 7 in the no RRT group

**Table 3:** Liver function test and bilirubin levels in ECMO patients according to cannulation type.

Type of ECMO Cannulation							
	Single-Site Avalon			Double-Site Standard			
	Day 1	Day 3	Day 7	Day 1	Day 3	Day 7	*p* *
TB (mg/dL)	2.63 ± 1.5 a	6.67 ± 3.5 b	9.5 ± 7.3 c	2.3 ± 1.3	2.40 ± 1.1	7.10 ± 4.9	0.25
AST (UI/L)	36.3 ± 8.7 #	57.6 ± 21.2 ##	98.6 ± 4.1	57.6 ± 21.2	26 ± 6.3	57 ± 31	0.24
ALT(UI/L)	30.6 ± 8.9 #	36 ± 21.8	37.3 ± 8.6	69 ± 66.6	29.3 ± 18.4	51.6 ± 30.7	0.33

Abbreviations: ECMO: extracorporeal membrane oxygenation; ALT: alanine transaminase; AST: aspartate transaminase; TB: total bilirubin level. a: *p* = 0.001 day 1 vs. day 3 in both groups; b: *p* = 0.009 day 3 vs. day 7 in both groups; c: *p* = 0.002 day 1 vs. day 7 in both groups; **#**: *p* = 0.01 day 1 vs. day 7 in the RRT group; **##**: *p*: 0.001 day 3 vs. day 7 in both groups. *p* * values refer to the ANOVA test used to evaluate the difference between the means of the three days of the two groups.

## Data Availability

The dataset generated and analyzed during the current study is available from the corresponding author on reasonable request.
